# Multi-window CT based Radiomic signatures in differentiating indolent versus aggressive lung cancers in the National Lung Screening Trial: a retrospective study

**DOI:** 10.1186/s40644-019-0232-6

**Published:** 2019-06-28

**Authors:** Hong Lu, Wei Mu, Yoganand Balagurunathan, Jin Qi, Mahmoud A. Abdalah, Alberto L. Garcia, Zhaoxiang Ye, Robert J. Gillies, Matthew B. Schabath

**Affiliations:** 1 Department of Radiology, Tianjin Medical University Cancer Institute and Hospital, National Clinical Research Center of Cancer, Key Laboratory of Cancer Prevention and Therapy, Tianjin’s Clinical Research Center for Cancer, Huanhuxi Road, Hexi District, Tianjin, 300060 China; 2 Department of Cancer Physiology, H. Lee Moffitt Cancer Center and Research Institute, 12902 Magnolia Drive, Tampa, FL 33612 USA; 3 Department of Epidemiology, H. Lee Moffitt Cancer Center and Research Institute, 12902 Magnolia Drive, Tampa, FL 33612 USA

**Keywords:** Lung cancer screening, Radiomics, NLST, Multi-window CT, Indolent lung cancer

## Abstract

**Background:**

We retrospectively evaluated the capability of radiomic features to predict tumor growth in lung cancer screening and compared the performance of multi-window radiomic features and single window radiomic features.

**Methods:**

One hundred fifty lung nodules among 114 screen-detected, incident lung cancer patients from the National Lung Screening Trial (NLST) were investigated. Volume double time (VDT) was calculated as the difference between continuous two scans and used to define indolent and aggressive lung cancers. Lung nodules were semi-automatically segmented using lung and mediastinal windows separately, and subtracting the mediastinal window region from the lung window region generated the difference region. 364 radiomic features were separately exacted from nodules using the lung window, the mediastinal window and the difference region. Multivariable models were conducted to identify the most predictive features in predicting tumor growth. Clinical information was also obtained from the database.

**Results:**

Based on our definition, 26% of the cases were indolent lung cancer. The tumor growth pattern could be predicted by radiomic models constructed using features obtained in the lung window, the difference region, and by combining features obtained in both the lung window and difference regions with areas under the receiver operator characteristic (AUROCs) of 0.799, 0.819, and 0.846, respectively. The multi-window feature model showed better performance compared to single window features (*P* < 0.001). Incorporating clinical factors into the multi-window feature models showed improvement, yielding an accuracy of 84.67% and AUROC of 0.855 for distinguishing indolent from aggressive disease.

**Conclusions:**

Multi-window CT based radiomics features are valuable predictors of indolent lung cancers and out performed single CT window setting. Combining clinical information improved predicting performance.

**Electronic supplementary material:**

The online version of this article (10.1186/s40644-019-0232-6) contains supplementary material, which is available to authorized users.

## Background

Lung cancer is the leading cause of cancer-related deaths among both men and women in the U.S. [1]. Screening and early detection of high-risk individuals, based on age and smoking history, can detect lung cancer at an earlier, more treatable stage, and has been shown to improve lung cancer survival rates [2, 3]. Specifically, the National Lung Screening trial (NLST) demonstrated a 20% reduction in lung cancer mortality among high-risk individuals screened with low-dose computerized tomography (LDCT) screening versus those screened with standard chest X-ray [4]. Based on the findings from the NLST, the U.S. Preventive Services Task Force issued a recommendation for annual lung cancer screening by LDCT [5].

Despite the mortality reduction benefit associated with lung cancer screening, there are concerns that a subset patients diagnosed with lung cancer in the screening setting may be due to overdiagnosis of slow growing, indolent cancer that may pose no threat and result in overtreatment [2, 6–9]. In the NLST, prior studies estimated that 18 to 22.5% of screen-detected cancers would not become symptomatic in a patient’s lifetime and would remain as indolent lung cancer [7]. Additionally, there have been several other screening studies that also estimated a range of indolent lung cancer rates to be between 2 and 25% [8–10]. Although the methodologies and cohort sizes may vary, the existence of indolent lung cancer in lung cancer screening poses an important public health concern. Overdiagnosis of indolent lung cancer results in additional, unnecessary screening, increased costs, higher levels of radiation exposures, undue stress for patients and their families, and unnecessary morbidity that is sometimes associated with overtreatment. Also, prior studies have shown that small indeterminate lung nodules (< 4 mm), which did not reach the criteria to be considered a positive screen in the NLST, that develop into lung cancer in subsequent screening intervals are associated with poorer survival and higher lung cancer mortality compared to those who had a baseline positive screen because of potentially aggressive growth in a relatively short amount of time (1 to 2 years) [11–13]. As CT imaging has an important role in the longitudinal clinical management of lung lesions, it is critical to find additional imaging-based biomarkers that could distinguish biologically indolent and aggressive lung cancer at an early stage of development and optimize the scan interval to reduce both overdiagnosis and underdiagnosis.

Radiomics has emerged as a powerful approach to characterize and quantify pulmonary nodules. By providing information on nodule size, shape, and spatial and temporal tumor heterogeneity, Radiomic features can be applied for risk prediction, diagnostic discrimination and disease progression [14–17]. Compared to conventional radiology practices based on visual interpretation, radiomics is the process of converting standard-of-care medical images into high-dimensional quantitative features that are mineable either by conventional biostatistical approaches or machine learning methods.

To date, few studies have been performed to investigate the association between radiomics and growth rate of lung nodules. Moreover, currently published radiomics work in lung nodules has focused on images acquired with single CT window, usually the lung window. Lee et al. [18] and Sajin et al. [19] showed that the different parts of lung nodules recognized by two CT windows (lung window and mediastinal window) were associated with different pathological components. In addition, some studies found that the ratio of disappearance tumor area between the mediastinal window setting and the lung window setting is related to clinical-pathologic characteristics and tumor aggressiveness and is a significant independent prognostic determinant for small lung adenocarcinoma [20, 21]. The motivation for our study comes from conventional radiology, which commonly cycles between both windows to improve diagnostic accuracy. Thus, we hypothesized that highly heterogeneous tumor with different morphology of lung cancer should be reflected with the use of different CT windows settings and multi-window CT based quantitative descriptors could provide an improved prospective clinical predictor for lung cancer screening. Therefore, we performed a radiomic analysis to identify image biomarkers to reveal differences between these two windows and to predict growth patterns of lung cancers in the lung cancer screening setting.

## Methods

### Study population

We obtained the LDCT images and clinical information for the NLST from the Cancer Data Access System (CDAS) [22]. The NLST study design, patient enrollment has have previously documented [4, 23, 24]. In brief, a total of 53,454 participants who are high-risk of lung cancer, with a smoking history of 30 pack years (former smokers or those who quit with less than 15 years) and 55 years or older were randomly assigned to LDCT or radiography examination and administered with baseline and two annual follow-up scans. Exclusion criteria included previous lung cancer history, undergoing chest CT within 18 months before enrollment and having an unexplained weight loss of more than 6.8 kg in the preceding year. If the lung cancer diagnosis was confirmed, the participants would be treated and left the following screening examination. This retrospective study was approved by the Institutional Review Board (IRB) at University of South Florida (USF) and informed consent was waived.

The present study used subset of patients that has been described in prior studies from our group [16, 25, 26]. Briefly, we identified 314 screen-detected, incident lung cancer patients, who were not diagnosed with lung cancer at baseline screening, but were diagnosed with lung cancer at either the first follow-up screening interval or second follow-up screening interval. These lung cancer cases were derived from prior published nested case-control studies described in [16, 26]. However, 200 cases were excluded for the following reasons: complete volumetric image sets were not available, the nodules at the baseline could not be identified using the location information provided by the publically available NLST data, and cases for which it is difficult to exactly contour the tumor margin at any CT window. As such, the final analytical cohort of incident lung cancer patients included 114 patients with 150 lesions. Among the 114 patients, 36 patients had imaging studies conducted for three time points (i.e., baseline, the first follow–up. and the second follow-up). Self-reported patient clinical data from the NLST used in this analysis were age at randomization, sex, pack-years smoked, family history of lung cancer, smoking status, and history of COPD.

### Volume-doubling time (VDT) and tumor growth patterns

Volume-doubling time (VDT) of a non-calcified nodule was used as the criteria for classifying indolent lung cancers versus aggressive lung cancers. Volumes were calculated at the baseline screen and all available follow-up screening intervals. And VDT for each nodule was calculated using the fowling equation:

$$ VDT=\frac{\ln 2\times {T}_i}{\ln \left({V}_i/{V}_o\right)} $$

Where *T*_*i*_ means interval time between two scans, *V*_0_ refers to the volume of the first scan, and *V*_i_ refers to the volume of the second scan.

Nodules with a VDT more than 400 days were classified as indolent/slow-growing lung cancer, and nodules with a VDT less than 400 days were classified as aggressive/fast-growing lung cancers.

### Tumor segmentation and Radiomic feature extraction

All lung nodules were reviewed and segmented by two clinical radiologists (H.L. and J.Q. with 15 and 12 years of experience in thorax imaging, respectively), who was aware of malignancy status but were blinded to clinical information and growth status. Lesions were identified and segmented using the Quantitative Imaging Decision Support (QIDS)® Platform (HealthMyne, Madison, WI) to delineate the tumor regions for this study. After identifying lesions and dragging the line along the longest diameter, a 2D delineation preview is presented to the user for editing or confirmation. Once confirmed the 2D delineation, a 3D segmentation is automatically performed, after which the boundaries can then be edited and confirmed. Manual editing occurred in about 8% of the nodule volumes because of pleural or fissure or vessel attachment. Each nodule was segmented under both standard lung window (window width 1500 Hu, window level, − 400 Hu) and mediastinal window (window width 400Hu, window level, 40Hu). All segmented images were reviewed by 2 radiologists in consensus and any discrepancies were discussed to reach consensus.

**Fig. 1:**
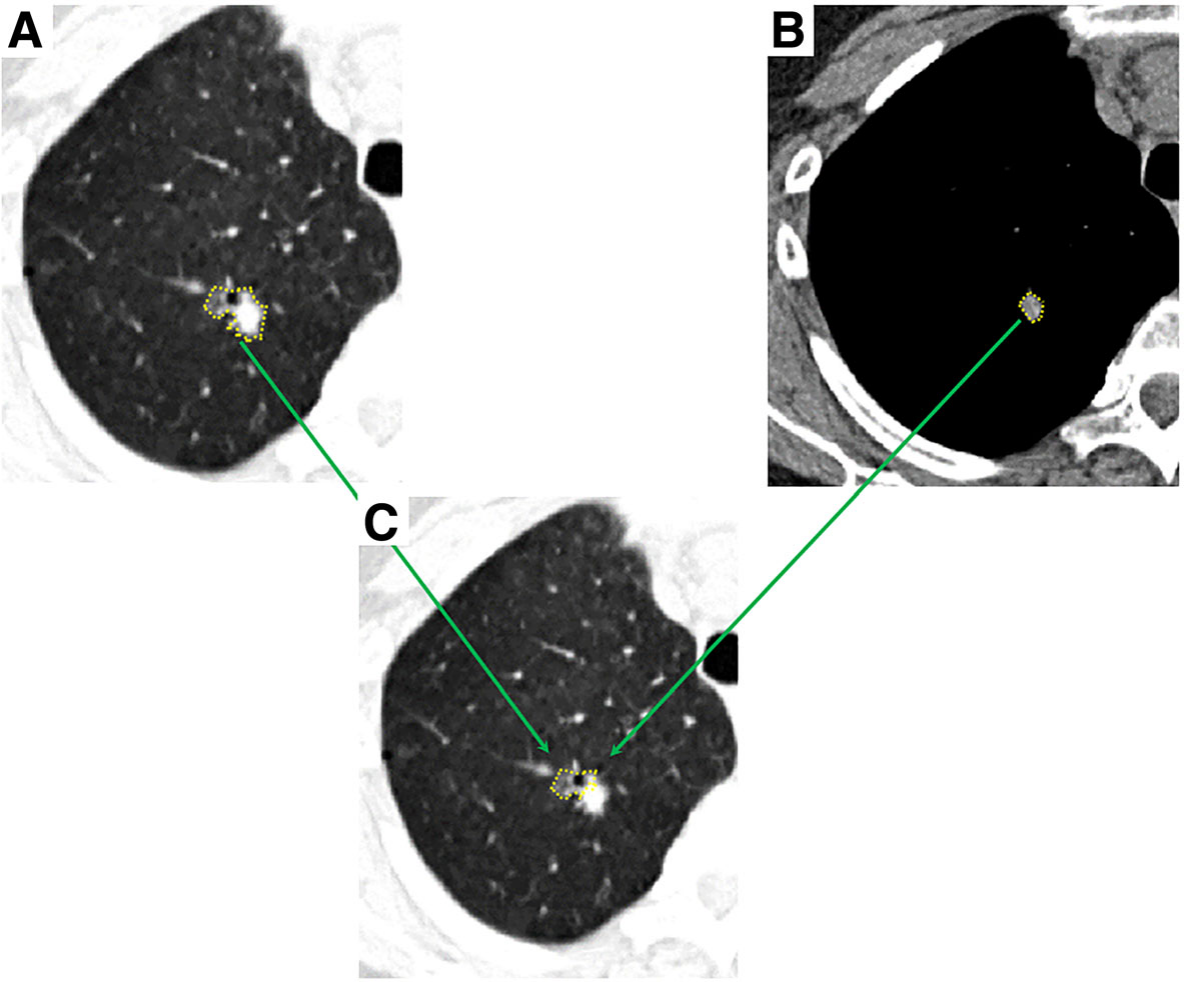
Difference region between lung window and mediastinal window settings. **a** Axial CT show an irregular part-solid nodule in the right upper lobe of lung in lung window. **b** The solid portion of the nodule showed in mediastinal window. **c** Based on two windows, the difference region can be obtained.

The two tumor masks (standard lung window mask and mediastinal window mask) were imported into MATLAB. The difference regions between the two windows (Fig. 1), voxels that appear in lung window but not the mediastinal window, were obtained and then radiomic features were obtained from the two different masks: standard lung window mask, difference region mask. Radiomics features were extracted using an in-house texture extractor implemented with MATLAB 2016b (MathWorks, Natick, USA). For each mask, 364 features were extracted, including 209 IBSI features according as previously described [27, 28], 125 Laws features and 30 wavelet features (Additional file 1: Table S1).

### Statistical analysis

To reduce the number of radiomic features, two separate dimensionality reductions were conducted. First, the Student’s t-test was performed for each feature comparing indolent lung cancers versus aggressive tumor. Statistically significant radiomic features (*p*-value < 0.05) were included. Next, the area under the receiver operating characteristic (AUROC) was calculated for each feature with Bootstrap resampling at 200X and features with a mean AUROC > = 0.5 were included. Radiomic features that were both statistically significant by the Student’s *t*-test and possessing an AUROC > = 0.05 were then tested for correlation using Pearson’s coefficient. Among correlated features that had a Pearson’s coefficient > =0.8, the feature with the largest mean AUROC was selected. The final features were then reduced using a backward elimination logistic regression approach (0.05 for entry and 0.10 for removal). Using this approach, three individual models were constructed using the lung window features, difference region features, and the combination of features derived from the lung window and the difference region. These were used to yield 3 distinct radiomics scores. Finally, we included patient information (sex and self-reported history of COPD) to the radiomics score based model to investigate the incremental complementary value to improve the predictors. All statistical tests were 2-sided. A *p*-value of less than 0.05 was considered statistically significant.

## Results

**Fig. 2:**
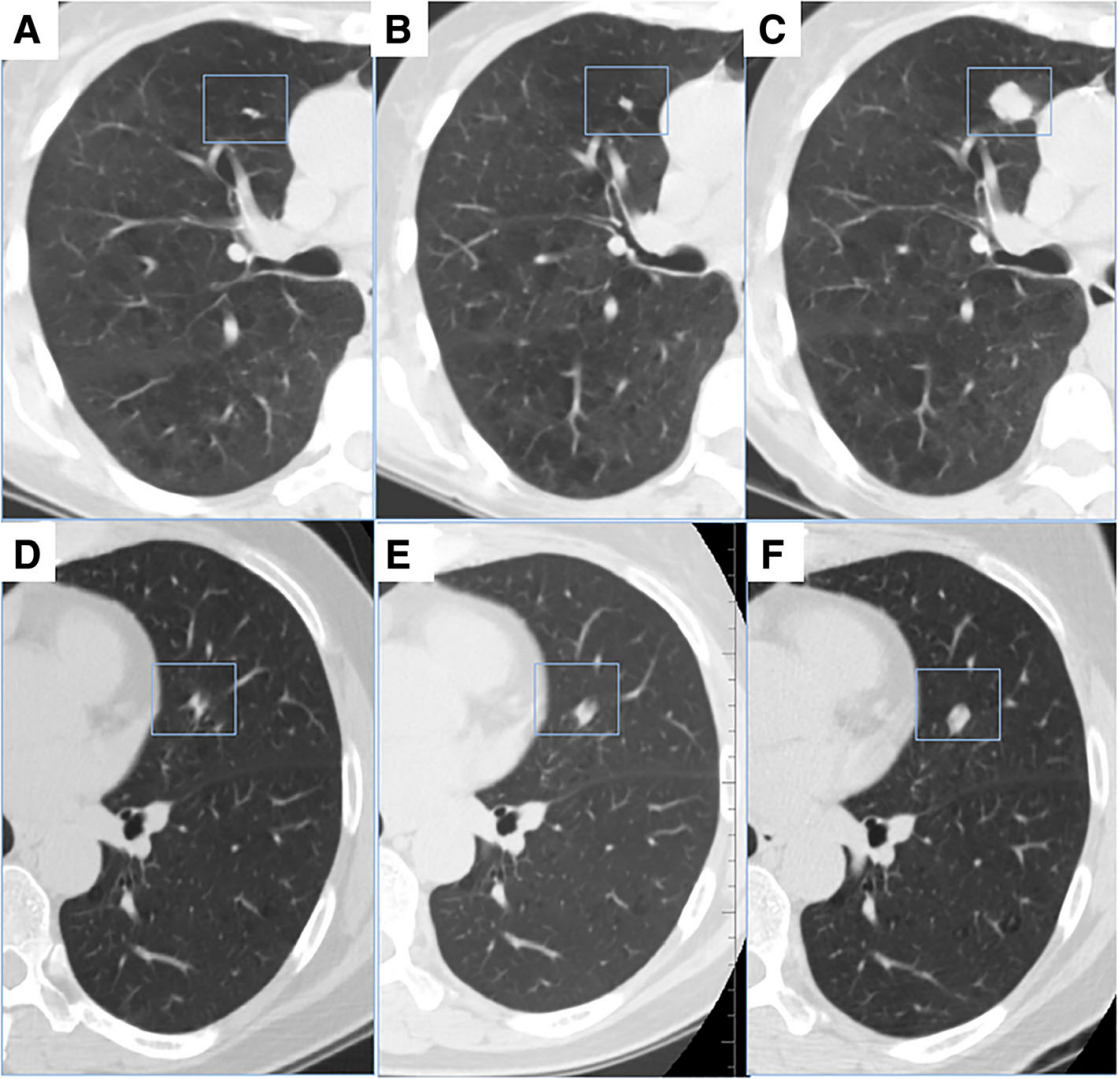
The lung cancers with mixed growth pattern during two round of follow up scan. **a-c** A nodule re-classified from indolent to aggressive. **a** Baseline scan (T0). Axial CT images show an irregular nodule in right upper lobe. **b** The first follow up (T1), with the interval days of 406 days and VDT 5713 days. **c** The second follow up (T2), with the interval days of 355 days and VDT 86 days. **d-f** A nodule re-classified from aggressive to indolent cancer**. d** Baseline scan (T0). Axial CT images show an amorphous nodule in left upper lobe. **e** The first follow up (T1), with the interval days of 430 days and VDT 114 days. **f** The second follow up (T2), with the interval days of 300 days and VDT 848 days

The patient demographic data are presented in Table 1. There were totally 39 (26%) nodules classified as indolent lung cancer (median VDT 583 days) compared to 111 (74%) nodules classified as aggressive (median VDT 148 days). There were 36 patients who had a baseline screening and two follow-up screens, among of which 17 patients exhibited mixed growth pattern during the two follow-up screening intervals. And 12 nodules from the first to second follow-up were re-classified from indolent to aggressive, while 5 nodules were re-classified from aggressive to indolent cancer (Fig. 2).

**Table 1:** Demographic characteristic of patients

Variable	Aggressive cancer (*n* = 77)	Indolent cancer (*n* = 20)	Mixed cancer (*n* = 17)	*P*
Age				0.196
Mean ± SD	65.29 ± 5.45	62.60 ± 4.71	65.18 ± 5.17	
Pack years smoked				0.704
Mean ± SD	65.01 ± 25.83	62.40 ± 18.77	51.43 ± 16.96	
Sex				0.006
Female	24 (31.17)	14(70.00)	9 (52.94)	
Male	53 (68.83)	6(30.00)	8 (47.06)	
Family history of lung cancer				0.386
Yes	20 (25.97)	6(30.00)	2 (11.76)	
No	57 (74.03)	14(70.00)	15 (88.24)	
Smoke status				0.309
Current	41 (53.25)	14(70.00)	8 (47.06)	
Former	36 (46.75)	6(30.00)	9 (52.94)	
History of COPD				0.035
Yes	16 (20.78)	0 (0)	1 (5.88)	
No	61 (79.22)	20 (100)	16 (94.12)	

Note: Data are presented as n, or n (%) Abbreviation: *COPD* chronic obstructive pulmonary disease

For the 77 patients with aggressive lung cancer, 16 of them have scans at 3 time points (baseline, the first follow up, the second follow up);For the 20 patients with indolent lung cancer, 2 of them have scans at 3 time points (baseline, the first follow up, the second follow up);For the 17 patients with mixed lung cancer, the nodules of 12 of them have indolent pattern at first but aggressive since the second scan, while the nodules of the rest 5 patients have aggressive pattern at first but indolent since the second scan

In our dataset, the volume of the nodule in lung window was in the range of 4.12~68.74 mm^3^, while the volume of the nodule in mediastinal window was in the range of 0~56.40 mm^3^. Volume was significantly different between the two groups, but was excluded at the final prediction model in the feature selection. There were significant differences in sex and self-reported COPD between indolent and aggressive lung cancers (Table 1). Female patients were much more likely to have indolent cancers (70.00% vs 31.17%) than male patients (*P* = 0.006). Concerning history of COPD, indolent lung cancers were more frequent in patients without history of COPD compared with aggressive lung cancers (*P* = 0.035). There were no differences in age (*P* = 0.196), pack-years smoked (*P* = 0.704), family history of lung cancer (*P* = 0.386), and smoking status (*P* = 0.309) between indolent and aggressive lung cancers. The AUROC of multivariable logistic regression model generated with the clinical features alone was 0.742(95% CI, 0.66 to 0.83), with accuracy of 62.00%, specificity of 54.05% and sensitivity of 84.62%.

**Fig. 3:**
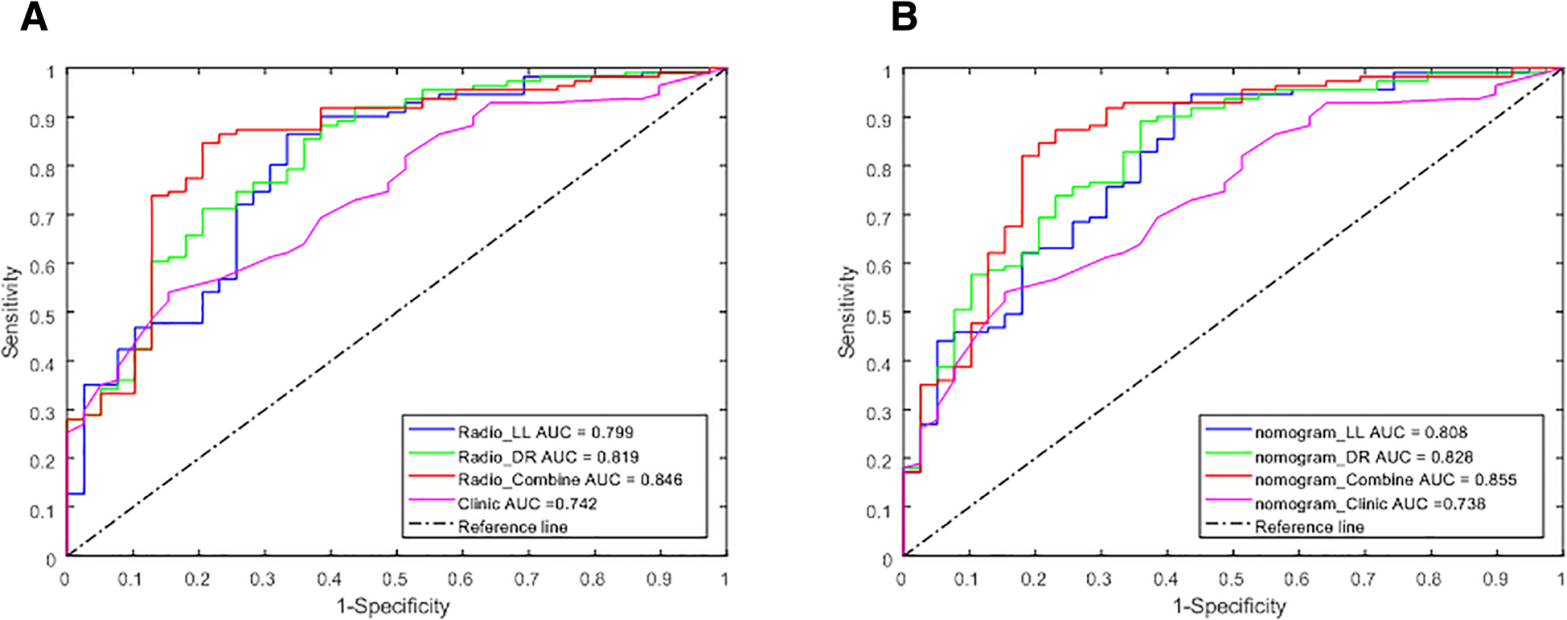
ROC curves for the prediction of tumor growth patterns obtained from 5000* bootstrap resampling. **a** Multivariable radiomics models **b** Nomogram models combing the radiomic features and clinical characteristics

**Fig. 4:**
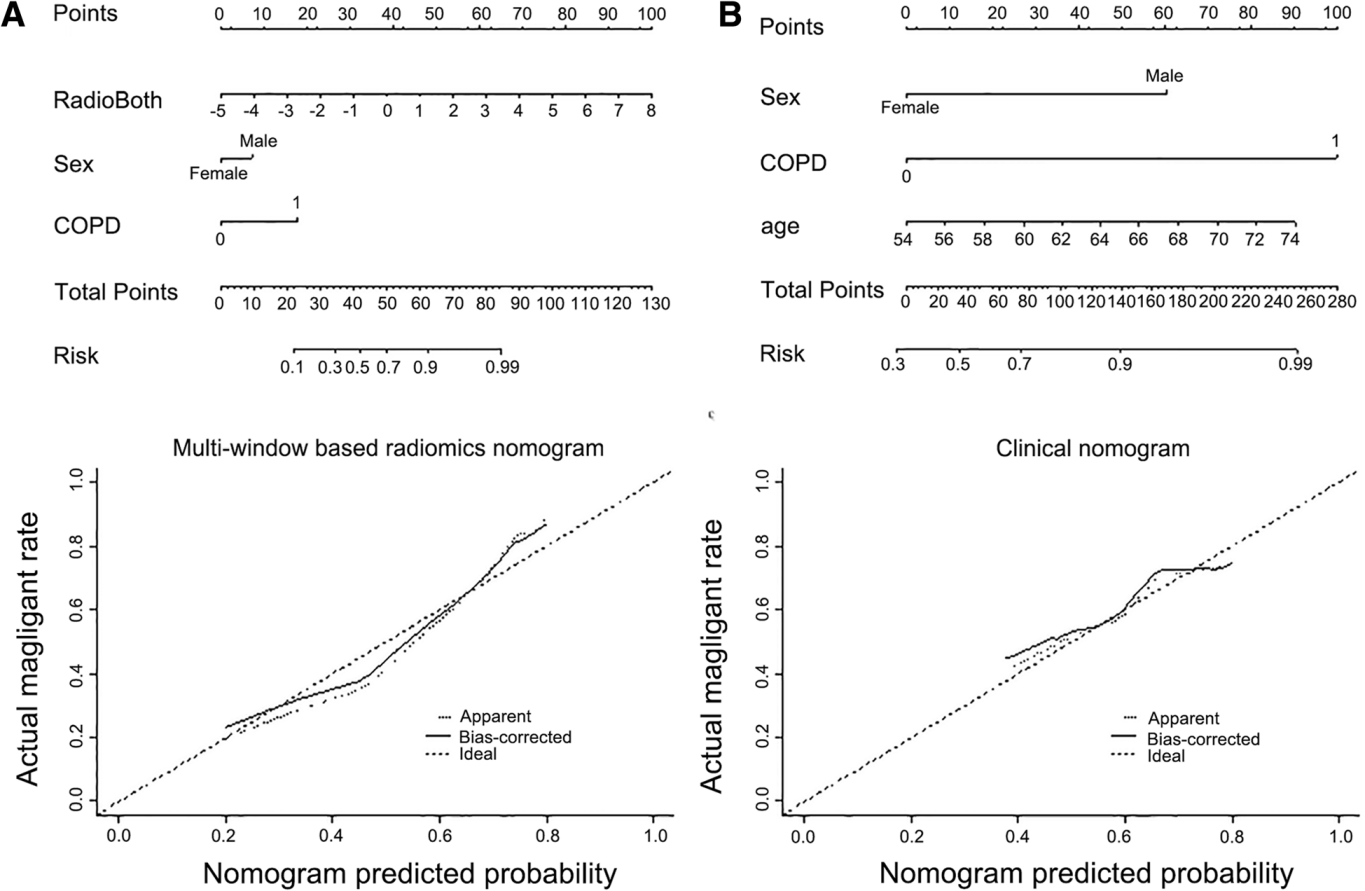
**a** The multi-window CT based Radiomics nomogram created with lung-window radiomic features and difference region radiomic features together. **b** The clinical nomogram created with clinical characteristics alone

The most informative radiomic features predicting lung cancer growth pattern were obtained from lung window and difference region between lung and mediastinal windows. The multivariable logistic regression model using radiomic features obtained in the difference region had better predictive power than the features from any single lung window (Table 2). The AUROC based on difference region features was 0.820 (95% CI, 0.74 to 0.90), with accuracy of 73.33%, specificity of 79.49% and sensitivity of 71.17%, while the AUROC based on single lung window features was 0.800 (95% CI, 0.72 to 0.88), with accuracy of 81.33%, specificity of 66.67% and sensitivity of 86.49%, When these two sets of features were combined, the AUROC was increased to 0.845 (95% CI, 0.77 to 0.92), with accuracy and sensitivity improved to 83.33 and 84.68%, respectively. Bootstrap re-sampling for internal validation was conducted and the odds and performance statistics did not change to a significant extent, with the AUROC based on difference region features, lung window features and combined these two settings features were 0.819 (95% CI, 0.742 to 0.90), 0.700 (95% CI, 0.72 to 0.88) and 0.846 (95% CI, 0.77 to 0.92), respectively (Table 2 and Fig. 3). We also report the improved incremental predictive value with the use of clinical information, which includes sex and history of COPD. The nomogram models generated with combined clinical and radiomic features (Fig. 3) were superior to the models created with radiomic features alone or clinical characteristic alone (Table 2 and Fig. 4).

**Table 2:** Multivariable models for the prediction of tumor growth speed

Covariate	Radiomics features			Radiomics features combined with demographics			
	mOR (95% CI)	Bootstrap mOR (95% CI)	Bootstrap *P* value	Covariate	mOR (95% CI)	Bootstrap mOR (95% CI)	Bootstrap *P* value
Lung Window Mask							
LW_F8	1.00 (1.00–1.01)	1.00 (1.00–1.01)	.004	Radio_LL	2.22 (1.51–3.28)	2.40 (1.38–4.16)	<.001
LW_F87	0.77 (0.67–0.88)	0.77 (0.67–0.88)	<.001	Sex	2.37 (0.99–5.68)	2.38 (0.91–6.22)	.073
LW_F311	1903.06 (4.87–7.44 × 10^5^)	1903.06 (4.87–7.44 × 10^5^)	.016	COPD	5.78 (0.68–49.04)	1.16 × 1016 (0–5.20 × 1056)	.018
AUC	0.800 (0.717–0.883)	0.799 (0.717–0.883)		AUC	0.813 (0.735–0.890)	0.808 (0.727–0.888)	
Accuracy	81.33%	81.33%		Accuracy	84.00%	84.00%	
Specificity	66.67%	66.67%		Specificity	58.97%	58.97%	
Sensitivity	86.49%	86.49%		Sensitivity	92.79%	92.79%	
Difference Region Mask							
DR_F8	1.01 (1.00–1.02)	1.01 (1.00–1.02)	<.001	Radio_DR	2.27 (1.56–3.31)	2.42 (1.45–4.03)	<.001
DR_F87	0.80 (0.70–0.92)	0.78 (0.64–0.96)	<.001	Sex	2.47 (1.00–6.08)	2.54 (0.95–6.81)	.055
DR_F286	0.89 (0.80–0.99)	0.89 (0.78–1.00)	.042	COPD	6.47 (0.72–58.03)	2.41 × 10^16^ (0–1.17 × 10^57^)	.006
DA_F311	836.55 (1.75–3.99 × 10^6^)	1499.14 (1.34–1.68 × 10^6^)	.038				
AUC	0.820 (0.743–0.896)	0.819 (0.742–0.896)		AUC	0.837 (0.767–0.907)	0.828 (0.753–0.903)	
Accuracy	73.33%	82.00%		Accuracy	84.00%	84.67%	
Specificity	79.49%	61.54%		Specificity	69.23%	69.23%	
Sensitivity	71.17%	89.19%		Sensitivity	89.19%	90.09%	
Combined Windows Mask							
LW_F44	66,379.49 (0.64–6.89 × 10^9^)	5.14 × 10^5^ (1.11–2.37 × 10^5^)	.020	Radio_Comb	2.31 (1.62–3.29)	2.49 (1.46–4.22)	<.001
LW_F311	1406.04 (2.53–7.83 × 10^5^)	2903.14 (1.66–5.06 × 10^6^)	.030	Sex	2.17 (0.88–5.35)	2.23 (0.85–5.84)	.008
DR_F8	1.01 (1.00–1.01)	1.01 (1.00–1.01)	.003	COPD	6.90 (0.76–62.40)	6.53 × 10^16^ (0–6.87 × 10^57^)	.017
DR_F87	0.80 (0.70–0.93)	0.78 (0.63–0.98)	.005				
DR_F296	0.85 (0.75–0.96)	0.83 (0.70–0.99)	.017				
AUC	0.845 (0.770 to 0.920)	0.846 (0.772 to 0.920)		AUC	0.861 (0.791 to 0.931)	0.855 (0.781 to 0.929)	
Accuracy	83.33%	84.00%		Accuracy	84.67%	84.67%	
Specificity	79.49%	79.49%		Specificity	76.92%	76.92%	
Sensitivity	84.68%	85.59%		Sensitivity	87.39%	87.39%	

Note. F8 means statistical 10th percentile, F19 means root mean square, F44 means volume at intensity fraction 90, F87 means weighted CoM_z, F296 means 3D Wavelet P1L2C10 feature, F311 means voxel dimension x, Radio_LL means radiomics score calculated by the selected lung window features; Radio_DR means radomics score calculated from the selected difference region features; Radio_comb means radiomics score calculated from the selected lung window and difference region features

NA, not available. These variables were eliminated in the multivariable logistic regression mode, so the OR and *P* values were not available

## Discussion

Using LDCT images and data from the NLST, we extracted radiomic features and calculated VDTs using a multi-window approach to identify features associated with tumor growth. Overall, radiomic features extracted from the combined window yielded a highly predictive model to discriminate indolent from aggressive lung cancers which yielded an AUROC of 0.85 and accuracy of 84.67%. The model derived from the combined window features resulted in better performance statistics compared to the models derived from the lung window and difference region only. Combining the most predictive radiomics features and demographic risk factors into a radiomics nomogram demonstrated the translation implication for individualized tumor growth speed estimation. As such, these data demonstrate that multi-window CT based radiomics features are valuable in improved personalization and precision screening and management of lung cancer.

Now that LDCT imaging is approved for screening and early detection of lung cancer, the implications of identified high rates of indolent cancers is a real-life concern. Bach [29] proposed a bipartite natural-history model of lung cancer, which classifies lung cancer into indolent versus aggressive as unique separate entities. However, the exact definition of indolent lung cancer is not uniform or consistent across studies. In NLST [7], indolent lung cancers were defined as the surplus set of cancers compared to standard chest radiography arm. In the Pittsburgh Lung Screening Study (PluSS) [10], Thalanayar et al. combined volume (VDT ≥ 400 days) and PET (maximal standardization uptake ≤1) information to define indolence and estimated a prevalence of 18.5%. Yankelevitz et al. [9] calculated the VDT (VDT ≥ 400 days) based on the size measurement of recorded in MLP (Mayo lung project) and MSK (the Memorial Sloan Kettering Cancer Center trial) studies to evaluate the indolent cases on chest radiography screening and 2 to 7% of indolence was identified. Using a similar definition, Lindell et al. [6] retrospectively evaluated the indolence in the LDCT screening of 5 years and reported a rate of 25%. In the Continuous Observation of Smoking Subjects (COSMOS) study [8], Veronesi et al. used VDT(VDT ≥ 400 days or 600 days) from volume to define indolent lung cancer or slow-growing, and suggested that cancer with a VDT of 400 days or more could be overdiagnosed.

Compared to the VDT from 2-dimention analysis, the VDT from 3-dimention has well reproducibility [30]. Volume changes estimated from the 2-dimention diameter may miss information of asymmetric growth [31]. Moreover, VDT has also significant association with lung cancer risk and lung cancer-specific mortality [8, 32]. Assessment of VDT was valuable in reducing false positives [33]. So VDT is a reliable and directive indicator of cancer aggressivity. In our study, using VDT from volumetric analysis as criteria, about 26% lesions were diagnosed as indolent lung cancer with median VDT 583 days, which were similar with previous report [6–8]. Recognizing these lung cancer with different growth pattern would be helpful in defining the time interval of following up to reduce the cost of screening and overtreatment for indolent lesions, at the same time, avoiding delaying the most better treatment opportunity for aggressive lung cancer.

In our analysis we found that 47% of the nodules exhibited inconsistent growth pattern between two time periods (i.e., baseline to first follow-up versus first follow-up to second follow-up), and 2 lesions became smaller in volume at some time point. Similar findings were also reported by previous studies [6, 34]. In Lindell’s [6] five-year lung cancer screening study, he reviewed the growth curves of 18 lung cancers with at least four times CT scans and found the growth appearance of lesions stratified with CT scan attenuation, survival and size were vary. He also found 4 tumors reduced during the follow up, including two bronchioloalveolar carcinoma and two non bronchioloalveolar carcinoma. Similarly, Leo [34] also reported a rare regression of lung cancer without any intervention. Classically, lung cancer evolution was according to the exponential growth model, but there is increasing evidence shows that the natural history of lung malignant nodules does not always fit this model. The complex interaction between stem cell and the microenvironment of the tumor and the immune system play an important role in tumor progression [35]. Our findings suggested the status evaluation of lung cancer at one time point may not always predict tumor growth and even mislead the lung nodule management. As such, non-invasive imaging-based predictors of tumor growth at different time point, as presented in our analysis, should be helpful to assist in identifying different growth pattern of lung cancer and selecting personalized follow up interval during lung cancer screening.

Although radiomics feature have been utilized in lung cancer risk prediction and diagnosis [14–16], our current analysis is the first to evaluate growth pattern of lung cancers using multi-window CT radiomic features. With the large amount of objective quantitative metrics extracted either from entire tumor or a particular interest of area within tumors, radiomics depict the intratumoral heterogeneity, which subjective radiologic descriptors are inadequate to capture, and are used to evaluate and monitor tumor cell evolution over time. However, most current quantitative metrics lack spatialness, especially for the lung LDCT scan, and most radiomics analysis of lung nodules are based on single lung window CT images. The spatially explicit analysis of tumor regions is a potential emerging key point of cancer imaging [36]. In the present study, we proposed “window” as a practical and objective way to define the lung tumor habitat spatially and extract radiomic features from lung window, mediastinal window and difference region between these two window settings separately. Although the most informative features in distinguishing indolent and aggressive lung cancer were from the lung window and the difference region (data not shown), the multi-window based difference region model had the better performance statistics (Table 2). Moreover, compared to the single lung window, the combined predictive model based on multi-window CT images resulted in statistically better performance, with the AUROC reached 0.85. The different CT window setting would play different role in describing lung cancer physiology; however, the relationships between quantitative imaging and pathology remains poorly understood to date. Some studies investigated that the solid portion of lung cancer in the mediastinal window was associated with the adenocarcinoma invasiveness and using mediastinal window setting criterion could improve the interobserver agreement in classifying the subsolid lung nodule [18, 19, 37]. Okada et al. [20] found the ratio of the tumor area of the mediastinal window to that of the lung window was prognostic. The 5-year survival was 48% in cases with a ratio of 0 to 25%, 87% with a ratio of 26 to 50%, 97% with a ratio of 51 to 75%, and 100% with a ratio of 76 to 100%. Moreover, the higher disappearance ratio of two CT window settings also related to less lymphatic, vascular vessel invasion, or nodal involvement. Thus, the difference region between lung window and mediastinal window showed the potential to identify the clinical-pathologic characteristics and aggressiveness of lung cancer. Our results support this conclusion. The mechanistic explanation for this observation is not known; however, the observation could be attributed to that most of the discrepancy region between two CT window settings are located in the peripheral of tumor, where the active regions of tumor stem cell are interacting with their surrounding microenvironment. Future work is needed to elucidate these findings and cumulatively these results provide further clues to explore the role of window-based radimoics features in improved personalization and precision medicine.

We also found that sex and history of COPD were significantly different between indolent lung cancer and aggressive lung cancer and that by including this information with the radiomics nomogram (shown in Fig. 4) improved prediction capabilities. As for sex-based difference in growth speed, our results were consistent with the following studies. Hasegawa et al. [38] revealed the mean VDT of lung tumor was longer in women (559 days for women and 387 days for men). Lindell et al. [6] got the greater difference between the sexes (688 days for women and 234 days for men) and thought the women had higher incidence of slow-growing or indolent lung cancer for histology type. The link between COPD and lung cancer has garnered substantial concerning over the past decade years and many epidemiological studies have consistently demonstrated an increased incidence of lung cancer in patients with history of COPD [39] [40]. The association between CDPD and tumor growth has little konwn, and our analyisis revealed that the incidence of COPD was lower in indolent lung cancer than that in agreesive lung cancer. This finding support the COSMOS study [6], which indicated that the slow-growing or indolent lung cancer was more common in low-risk persons.

We acknowledge some limitations of this analysis. First, the sample size was modest because of strict inclusion criteria. Also, we did not stratify the lung nodules according to the attenuation, because the discrepancy between the two CT window settings had already included the density information. Next, the participants of NLST were from different U.S. medical centers and the CT scanning parameters were not consistent, however, which would be the superiority for the extracted features to generalize to other screening or incidentally-detected lung cancer cohort. Although we performed backward-elimination bootstrapping for internal validation of our final models, further independent validation cohort across institutions would be helpful to confirm these findings.

## Conclusions

In conclusion, we have found that the multi-window CT based quantitative radiomic signatures showed the potential to reveal and predict the tumor growth speed non-invasively, and could identify the indolent subgroup from aggressive lung cancer, thus, would be valuable for precision lung cancer screening and longitude management of lung cancer.

## Additional file

Additional file 1: **Table S1.** 125 Laws features and 30 wavelet features. (DOCX 40 kb)

## Data Availability

The datasets used and analysed during the current study are available from the corresponding author on reasonable request.

## Abbreviations

AUROC: Area under the receiver operating characteristic
CDAS: Cancer Data Access System
LDCT: Low-dose computerized tomography
NLST: National Lung Screening trial
QIDS: Quantitative Imaging Decision Support
USF: University of South Florida
