# Supplementary material for: Multi-window CT based Radiomic signatures in differentiating indolent versus aggressive lung cancers in the National Lung Screening Trial: a retrospective study
Source: Cancer Imaging. 2019 Jun 28;19:45. doi: 10.1186/s40644-019-0232-6 (PMC6599273; doi:10.1186/s40644-019-0232-6)
Supplement: Supplementary file 1 — Table S1. 125 Laws features and 30 wavelet features. (DOCX 40 kb) [file 40644_2019_232_MOESM1_ESM.docx]

**Table S1: 125 Laws features and 30 wavelet features**

| **Laws features** | Laws features are constructed from a set of five one-dimensional filters each designed to reflect to a different type of structure in the image. These one-dimensional filters are defined as E5 (edges), S5 (spots), R5 (ripples), W5 (waves), and L5 (low pass, or average gray value). By combining any three of the above 1-D convolution filters, we could obtain 125 3-D filters, and generated 125 filtered images. | For each filtered images, the energy was calculated. $Energy=\frac{1}{M\times N\times L}\sum_{i}^{M} \sum_{j}^{N} \sum_{k}^{L} h^{2}\left(i,j,k\right)$   Where M, N and L are filtered images dimensions, $h(i,j,k)$ means the filtered image. |
| --- | --- | --- |
| **Wavelet features** | The discrete wavelet transform can iteratively decompose an image (3D) into four components. Each iteration splits the image both horizontally and vertically into low-frequency (low pass) and high frequency (high pass) components. Thus, four components are generated: a high-pass/high-pass component consisting of mostly diagonal structure, a high-pass/low-pass component consisting mostly of vertical structures, a low-pass/high-pass component consisting mostly of horizontal structure, and a low-pass/low-pass component that represents a blurred version of the original image. Subsequent iterations then repeat the decomposition on the low-pass/low-pass component from the previous iteration. | For each component, we calculated the energy feature. $Energy=\frac{1}{M\times N\times L}\sum_{i}^{M} \sum_{j}^{N} \sum_{k}^{L} h^{2}\left(i,j,k\right)$   Where M, N and L are the dimensions of each subblock, $h(i,j,k)$ means the subblock elements. |
